# Testing the Geroscience Hypothesis in Lower Urinary Tract Symptoms of Aging

**DOI:** 10.1093/geroni/igaf122.404

**Published:** 2025-12-31

**Authors:** Iman Al-Naggar

**Affiliations:** UConn Health, Farmington, Connecticut, United States

## Abstract

Aging and the metabolic syndrome—a condition that accelerates biological aging—are both major risk factors for lower urinary tract symptoms (LUTS). LUTS, including urinary frequency, urgency, incontinence, and nocturia, substantially reduce quality of life and contribute to depression, social isolation, falls, fractures, and loss of independence in older adults. Dysfunction involving the brain-to-bladder axis presents as declines in physiological resilience, and existing therapies such as anticholinergics targeting detrusor smooth muscle provide limited benefit and are often discontinued due to side effects. Recent evidence implicates fundamental biological mechanisms of aging—chronic inflammation, mitochondrial dysfunction, and oxidative stress—in the pathogenesis of metabolic syndrome–associated LUTS. Preclinical studies in mouse models of aging and metabolic syndrome demonstrate increased bladder dysfunction, supporting these mechanisms as potential therapeutic targets. This presentation will describe the rationale and design of an ongoing pilot clinical trial testing MitoQ, a mitochondria-targeted antioxidant with gerotherapeutic properties, for ability to alleviate LUTS in older women with metabolic syndrome. By moving from animal models to human trials, this work aims to test whether targeting hallmarks of aging can modify urologic symptoms. The talk will also highlight key design considerations and practical challenges in translating the geroscience hypothesis into clinical research.

